# Association of Sleep Duration with Weight Gain and General and Central Obesity Risk in Chinese Adults: A Prospective Study

**DOI:** 10.1002/oby.22713

**Published:** 2019-12-26

**Authors:** Xuejuan Ning, Jun Lv, Yu Guo, Zheng Bian, Yunlong Tan, Pei Pei, Junshi Chen, Shichuan Yan, Huimei Li, Zhifang Fu, Yiping Chen, Huaidong Du, Zhengming Chen, Canqing Yu, Liming Li, for the China Kadoorie Biobank (CKB) Collaborative Group

**Affiliations:** ^1^ Department of Epidemiology & Biostatistics School of Public Health Peking University Beijing China; ^2^ Chinese Academy of Medical Sciences Beijing China; ^3^ China National Center for Food Safety Risk Assessment Beijing China; ^4^ Department of NCDs Prevention and Control Heilongjiang CDC Harlin Heilongjiang China; ^5^ Department of NCDs Prevention and Control Meilan CDC Haikou China; ^6^ Clinical Trial Service Unit and Epidemiological Studies Unit (CTSU) Nuffield Department of Population Health University of Oxford Oxford UK

## Abstract

**Objective:**

Evidence on the association between sleep duration and obesity among adults is inconsistent. Prospective studies investigating the association in Chinese adults have been limited. This study aims to prospectively evaluate sleep duration in relation to subsequent weight gain and general and central obesity risk among Chinese adults.

**Methods:**

A total of 21,958 participants aged 30 to 79 years reported their daily sleep duration. Obesity indicators were objectively measured; then significant weight gain (≥ 5 kg) and general and central obesity were modeled as the outcome. Logistic regression models were used to estimate odds ratios and 95% CIs.

**Results:**

Average sleep duration was 7.5 hours at baseline. During 8.0 ± 0.8 years of follow‐up, participants who reported sleeping ≤ 6 hours had higher risk for significant weight gain than those who slept 7 hours (multivariable‐adjusted odds ratio: 1.13; 95% CI: 1.02‐1.29). The association was stronger among those who were physically inactive at baseline (*P* = 0.04 for interaction). Short sleep duration was also associated with subsequent incident central obesity, with odds ratio of 1.13 (95% CI: 1.00‐1.28), but not with incident general obesity (*P* = 0.31).

**Conclusions:**

Compared with those who slept 7 hours per day, short sleepers had an increased risk of significant weight gain and central obesity.

**Study Importance**

What is already known?

- Previous studies, which are mostly cross‐sectional studies, have suggested that short sleep duration is associated with weight gain.
- Existing prospective observational studies have reported mixed results.
- Therefore, it remains unclear whether short sleep duration is associated with a higher risk for obesity and whether there exists a U‐shaped relationship in which long sleep duration is also associated with higher risk for obesity.
- In addition, large prospective studies that have examined the association of sleep duration with central obesity in middle‐aged and older adults are scarce.

What does this study add?

- Our results suggest that short sleep duration, one of products of fast‐paced life, is associated with gaining weight significantly, and the weight gain is likely to be accrued on the abdomen.
- To our knowledge, this is by far the largest prospective study reporting a negative association between sleep duration and central obesity in healthy adults who are free of common comorbidities.
- Our findings emphasize the important role of short sleep duration in weight control and central obesity management.

## Introduction

Obesity has been a major public health issue worldwide and has nearly tripled in recent decades 1. According to the Global Burden of Disease Study, the level of BMI has increased significantly over the past decade, and elevated BMI ranked as the fourth leading risk factor that caused 4.72 million deaths and 148 million disability‐adjusted life‐years in 2017 2. Meanwhile, average sleep duration has been decreasing significantly because of lifestyle changes, mostly attributing to caffeine consumption, cigarette smoking, sleeping pattern, etc. 3. However, adequate sleep is of great significance for individuals at all ages. Evidence has shown that sleep deprivation could have negative influences on our nervous system, endocrine system, and cardiovascular system 3.

The potential link of sleep duration with obesity has been explored for years. However, large prospective studies conducted in the Chinese population have been lacking. Considering the Chinese population is currently in the process of fast socioeconomic growth and rapid nutritional transition, it is of importance to examine how short sleep duration, accompanied by the fast pace of life, might synergize with a potential increasing obesity trend. On the other hand, although several cross‐sectional studies have suggested a link between short sleep duration and the risk of obesity in adults 4, 5, 6, 7, 8, 9, it is difficult to establish a temporal relationship because of the nature of a cross‐sectional study design. Prospective observational studies still have not been able to give a clear answer, as some suggest a negative relationship 10, 11, 12, 13, 14, 15, some suggest a potential U‐shaped relationship 16, 17, and others suggest no association 18, 19, 20. In addition, central obesity, the type of obesity shown to explain more of the obesity‐related health risk 21, has been related to short sleep duration in cross‐sectional studies 22; however, the results were still inconsistent. Therefore, the present study aims to examine the prospective association between sleep duration and the risk of weight gain and different types of obesity in Chinese adults.

## Method

### Participants

The present study was based on the China Kadoorie Biobank, which enrolled 500,000 adults aged 30 to 79 years in 10 survey areas (5 urban and 5 rural). Detailed information about the study design has been reported elsewhere 23, 24. Briefly, the baseline survey was conducted from 2004 to 2008, which comprehensively collected questionnaires and blood samples and implemented physical examination. The second resurvey was undertaken during 2013 and 2014, which involved 5% (*n* = 24,996) of randomly chosen surviving participants in the cohort.

The present study included participants who attended both the baseline survey and the second resurvey. The follow‐up time started from the date they were enrolled at baseline (2004‐2008) until the date of the second resurvey (2013‐2014). We excluded those who had missing values on critical variables, such as BMI (*n* = 171) and waist circumference (WC) (*n* = 3); those who had coronary heart diseases (*n* = 714), stroke (*n* = 223), cancer (*n* = 102), and chronic obstructive pulmonary disease (*n* = 1,637); and those who tried to reduce weight in the year prior to baseline (*n* = 255), leaving a total of 21,958 participants in the analysis of sleep duration with significant weight gain. As for the analysis of the association with incident general and central obesity, we further excluded participants with general obesity (*n* = 2,320) or central obesity (*n* = 7,150) at baseline, leaving 19,638 and 14,808 participants for each of the analyses, respectively.

The Ethical Review Committee of the Chinese Center for Disease Control and Prevention (Beijing, China) and the Oxford Tropical Research Ethics Committee, University of Oxford (UK), approved the study.

### Obesity indexes

Physical examination at both surveys was performed by trained health workers following standardized procedures. Height was measured in centimeters using unified height meters and was reserved to 0.1 cm. Weight was measured with coat, shoes, and socks off, using Tanita TBF‐300GS constitution analyzer (Tanita, Tokyo, Japan). The weight of clothes was subtracted according to different seasons. WC was measured using standard tape. Weight gain was defined as the increase of weight between baseline and second resurvey. A weight gain ≥ 5 kg was considered gaining weight significantly, as it has been used in previous studies 15, 25, and 5 kg was suggested to be a reasonable weight change caused by lifestyle modification 26. BMI was calculated as weight (kilograms) divided by height (meters squared). General obesity was defined as BMI ≥ 28.0 27. Central obesity was defined as WC ≥ 90 cm in males and WC ≥ 80 cm in females according to International Diabetes Federation cutoffs for South Asians, Chinese, and Japanese 28.

### Sleep duration

At the baseline survey, participants were asked to report the number of hours they slept a day during the last 12 months. The question was phrased as “On average, how many hours of sleep do you get in a 24‐hour (h) period (including naps)?’’ Respondents could report in only 1‐hour increments. According to the American National Sleep Foundation’s sleep time recommendation, short sleep duration was defined as sleeping 6 hours or less, and long sleep duration was defined as sleeping 9 hours or more 29. Therefore, we divided sleep duration into the following four groups: ≤ 6 hours, 7 hours, 8 hours, and ≥ 9 hours.

### Covariates

A laptop‐based questionnaire at baseline survey was administered by trained interviewers to collect information on sociodemographic factors, lifestyle, and health status. Sociodemographic factors included age, sex, study region, education level (primary school and below, high school, technical school/college and above), and marital status (married, not married); lifestyle factors included smoking (never smoker, occasional smoker, ex‐regular smoker, smoker), alcohol drinking (never regular drinker, ex‐regular drinker, occasional or seasonal drinker, monthly drinker, reduced intake drinker, weekly drinker), tea drinking (never or almost never, occasionally, at least once a week), diet frequency (daily, 4‐6 days per week, 1‐3 days per week, monthly, never/rarely), and physical exercise (metabolic equivalent task hours/day); and health status included snoring habit (frequently, sometimes, don’t know), depression (no, mild, major depression), self‐rated health (excellent, good, fair, poor), and history of diabetes.

### Statistical analyses

We described baseline characteristics by sleep duration. Means or percentages were adjusted for age, sex, and study region using general linear models or logistic models. Incidences of significant weight gain, general obesity, and central obesity were compared across sleep duration groups adjusting for age, sex, and study region. Multiple logistic regression models were used to estimate odds ratios (OR) and 95% CIs to examine the association of sleep duration with obesity. Model 1 adjusted for age and sex. Further adjustment in model 2 included study region, education level, marital status, smoking, alcohol drinking, tea drinking, diet frequency, physical exercise, snoring, depression, and health rating. Model 3 additionally adjusted for history of diabetes, baseline weight and height, and BMI or WC.

Subgroup analyses according to baseline characteristics were conducted to explore the modification effects of these characteristics on the association between sleep duration and weight gain. Multiplicative interactions were tested by putting interaction terms in the fully adjusted models (model 3). To test the robustness of the association, a series of sensitivity analyses were performed by applying the following additional exclusion criteria: reported sleep difficulties (difficulties in initiating/maintaining sleep, early morning awakening, daytime dysfunction) at baseline, diabetes at baseline, diagnosed depression at second resurvey, reported coffee drinking at second resurvey, and newly diagnosed stroke or transient ischemic attack cases during the follow‐up period. All analyses were conducted using SAS version 9.3 (SAS Institute, Inc., Cary, North Carolina), and statistical significance was indicated by two‐tailed *P* < 0.05.

## Results

Among the 21,958 participants at baseline, average sleep duration was 7.5 hours; 20.8% of participants reported sleeping ≤ 6 hours, while 17.7% reported sleeping ≥ 9 hours. Participants who had shorter sleep durations were more likely to be older, living in urban areas, unmarried, and physically active but less likely to be snoring or drinking tea (Table 1).

**Table 1:** Baseline characteristics of study participants (*n* = 21,958) by sleep duration groups

	Average sleep duration (h)^b^	Sleep duration^a^, ^b^			
		≤ 6 hours	7 hours	8 hours	≥ 9 hours
***n***		4,559	5,357	8,155	3,887
**Region**					
**Urban**	7.3	2,328 (25.0)	2,794 (30.0)	3,160 (34.0)	1,023 (11.0)
**Rural**	7.7	2,231 (17.6)	2,563 (20.3)	4,995 (39.5)	2,864 (22.6)
**Sex**					
**Male**	7.4	1,689 (20.4)	2,069 (25.0)	3,124 (37.8)	1,392 (16.8)
**Female**	7.4	2,870 (20.8)	3,288 (24.0)	5,031 (61.7)	2,495 (18.2)
**Age (y)**					
**30‐44**	7.7	1,038 (14.2)	1,727 (23.6)	3,043 (41.6)	1,511 (20.6)
**45‐59**	7.4	2,349 (22.4)	2,611 (24.9)	3,787 (36.1)	1,735 (16.6)
**60‐78**	7.2	1,172 (28.2)	1,019 (24.5)	1,325 (31.9)	641 (15.4)
**Education level**					
**Below high school**	7.4	3,701 (20.8)	4,118 (23.2)	6,612 (37.2)	3,351 (18.8)
**High school and above**	7.4	858 (20.6)	1,239 (29.7)	1,543 (37.0)	536 (12.8)
**Marriage status**					
**Not married**	7.2	516 (32.3)	404 (25.3)	463 (29.0)	214 (13.4)
**Married**	7.5	4,043 (19.9)	4,953 (24.3)	7,692 (37.8)	3,673 (18.0)
**Smoking**					
**Occasional smoker/nonsmoker **	7.4	3,233 (20.8)	3,762 (24.3)	5,726 (36.9)	2,791 (18.0)
**Ex-regular smoker/smoker**	7.5	1,326 (20.6)	1,595 (24.7)	2,429 (37.7)	1,096 (17.0)
**Alcohol drinking**					
**Not weekly**	7.4	3,860 (20.6)	4,526 (24.1)	7,008 (37.4)	3,365 (17.9)
**Weekly**	7.4	699 (21.9)	831 (26.0)	1,147 (35.9)	522 (16.3)
**Tea drinking**					
**Not weekly**	7.4	3,170 (21.3)	3,659 (24.6)	5,414 (36.5)	2,612 (17.6)
**Weekly**	7.5	1,389 (19.6)	1,698 (23.9)	2,741 (38.6)	1,275 (18.0)
**Snoring**					
**No/don’t know**	7.4	2,449 (20.8)	2,768 (23.4)	4,414 (37.4)	2,174 (18.4)
**Yes**	7.5	2,110 (20.8)	2,589 (25.5)	3,741 (36.9)	1,713 (16.9)
**Physical activity level, MET**					
**Low**	7.5	1,599 (23.2)	1,607 (23.3)	2,379 (34.5)	1,305 (18.9)
**Medium**	7.5	1,544 (20.9)	1,806 (24.4)	2,727 (36.9)	1,312 (17.8)
**High**	7.3	1,416 (18.4)	1,944 (25.3)	3,049 (39.7)	1,270 (16.5)

a Shown as numbers and adjusted percentages in parentheses.

b Averaged sleep durations and percentages adjusted for age, regions, and sex as appropriate.

MET, metabolic equivalent for task.

During 8.0 ± 0.8 years of follow‐up, 3,159 (14.4%) participants had significant weight gain. Among 19,638 participants without general obesity at baseline, 5.5% developed incident general obesity; the corresponding incidence of central obesity was 24.6% among 14,808 participants without central obesity at baseline.

Compared with those who slept 7 hours, short sleepers had a significantly higher risk for significant weight gain in model 1 (OR = 1.15 [95% CI: 1.02‐1.29]), and the association did not change after further adjusting for potential confounders (Table 2). Such associations were generally homogeneous according to subgroup analyses by most of the baseline characteristics (Table 3), except that short sleepers who were relatively physically inactive were at higher risk of gaining weight (*P*_ _= 0.04 for interaction), with OR = 1.34 (95% CI: 1.07‐1.67).

**Table 2:** Adjusted obesity incidences and odds ratios (95% CI) of obesity by sleep duration

	Sleep duration			
	≤ 6 hours	7 hours	8 hours	≥ 9 hours
***Significant (≥ 5 kg) weight gain***				
***n* = 21,958**	4,559	5,357	8,155	3,887
**Average weight gain (kg)**	0.969	0.772	0.794	0.738
**Significant weight gain (%)** a	14.5	12.8	12.7	13.7
**Model 1** b	1.15 (1.02‐1.29)*	1 (ref)	1.01 (0.91‐1.11)	1.10 (0.98‐1.24)
**Model 2** c	1.14 (1.02‐1.29)*	1 (ref)	1.00 (0.90‐1.10)	1.08 (0.96‐1.22)
**Model 3** d	1.15 (1.02‐1.29)*	1 (ref)	0.99 (0.89‐1.10)	1.08 (0.96‐1.22)
***General obesity***				
***n* = 19,638**	4,061	4,750	7,339	3,488
**Cumulative incidence (%)** a	5.4	5.0	5.2	5.1
**Model 1** b	1.08 (0.89‐1.30)	1 (ref)	1.03 (0.88‐1.21)	1.02 (0.84‐1.24)
**Model 2** c	1.09 (0.90‐1.32)	1 (ref)	1.05 (0.89‐1.24)	0.99 (0.81‐1.21)
**Model 3** d	1.11 (0.91‐1.35)	1 (ref)	1.04 (0.87‐1.23)	0.97 (0.79‐1.20)
***Central obesity***				
***n* = 14,808**	3,008	3,547	5,609	2,644
**Cumulative incidence (%)** a	34.5	32.5	33.4	33.6
**Model 1** b	1.10 (0.99‐1.22)	1 (ref)	0.99 (0.90‐1.08)	0.99 (0.89‐1.10)
**Model 2** c	1.12 (1.00‐1.24)*	1 (ref)	1.03 (0.94‐1.13)	1.02 (0.91‐1.15)
**Model 3** d	1.13 (1.00‐1.28)*	1 (ref)	1.00 (0.90‐1.11)	1.03 (0.90‐1.17)

a Adjusted for age, sex, and region.

b Adjusted for age and sex.

c Additionally adjusted for region, education, marital status, smoking, alcohol drinking, physical activity, diet, tea drinking, health rating, snoring, and depression.

d Additionally adjusted for diabetes, baseline weight and height, baseline weight circumference, or baseline BMI.

* *P* < 0.05.

**Table 3:** Adjusted odds ratios (95% CI) of weight gain ≥ 5 kg by sleep duration and subgroups of baseline characteristics among 21,958 participants

	Sleep duration^a^				
	≤ 6 hours	7 hours	8 hours	≥ 9 hours	*P*_interaction_
***n***	4,559	5,357	8,155	3,887	
**Age**					0.21
**< 50**	1.12 (0.92‐1.35)	1 (ref)	1.06 (0.92‐1.23)	1.12 (0.94‐1.33)	
**50‐59**	1.12 (0.94‐1.34)	1 (ref)	0.99 (0.84‐1.17)	1.07 (0.87‐1.31)	
**≥ 60**	1.13 (0.83‐1.54)	1 (ref)	0.69 (0.50‐0.96)	0.89 (0.61‐1.30)	
**Sex**					0.76
**Male**	1.11 (0.93‐1.33)	1 (ref)	1.04 (0.89‐1.21)	1.08 (0.89‐1.31)	
**Female**	1.17 (1.00‐1.37)	1 (ref)	0.95 (0.83‐1.09)	1.05 (0.90‐1.24)	
**Smoking**					0.88
**Occasional smoker/nonsmoker**	1.13 (0.98‐1.31)	1 (ref)	0.93 (0.82‐1.05)	1.03 (0.89‐1.20)	
**Ex-regular smoker/smoker**	1.16 (0.95‐1.43)	1 (ref)	1.11 (0.93‐1.32)	1.14 (0.92‐1.41)	
**Alcohol drinking**					0.60
**Not weekly**	1.14 (1.00‐1.30)	1 (ref)	0.97 (0.87‐1.09)	1.05 (0.91‐1.20)	
**Weekly**	1.13 (0.85‐1.51)	1 (ref)	1.07 (0.83‐1.38)	1.22 (0.91‐1.66)	
**MET (h/d)**					0.04
**< 12.6**	1.34 (1.07‐1.67)	1 (ref)	0.92 (0.75‐1.14)	1.06 (0.84‐1.34)	
**12.6‐25.2**	1.04 (0.84‐1.28)	1 (ref)	0.90 (0.75‐1.07)	0.89 (0.71‐1.10)	
**≥ 25.3**	1.08 (0.89‐1.32)	1 (ref)	1.11 (0.95‐1.30)	1.28 (1.06‐1.55)	
**Diabetes**					0.12
**Yes**	0.79 (0.41‐1.54)	1 (ref)	0.66 (0.33‐1.29)	0.71 (0.32‐1.58)	
**No**	1.16 (1.03‐1.31)	1 (ref)	1.01 (0.91‐1.12)	1.10 (0.97‐1.24)	
**Baseline BMI (kg/m^2^)**					0.20
**< 18.5**	1.12 (0.61‐2.06)	1 (ref)	0.73 (0.41‐1.29)	0.73 (0.37‐1.46)	
**18.5‐23.9**	1.02 (0.87‐1.20)	1 (ref)	0.95 (0.83‐1.09)	0.98 (0.84‐1.15)	
**24.0‐27.9**	1.23 (0.99‐1.53)	1 (ref)	1.02 (0.84‐1.24)	1.20 (0.95‐1.50)	
**≥ 28.0**	1.76 (1.16‐2.70)	1 (ref)	1.44 (0.98‐2.12)	1.55 (1.00‐2.42)	
**Baseline WC**					0.09
**Male < 90 cm, female < 80 cm**	1.10 (0.96‐1.27)	1 (ref)	0.96 (0.85‐1.08)	1.04 (0.90‐1.19)	
**Male > 90 cm, female > 80 cm**	1.29 (1.02‐1.62)	1 (ref)	1.07 (0.87‐1.32)	1.23 (0.96‐1.56)	

a Adjusted for age, sex, region, education, marital status, smoking, alcohol drinking, physical activity, diet, tea drinking, health rating, snoring, depression, diabetes, baseline weight, and baseline height as appropriate.

Although a higher incidence of general obesity among short sleepers was observed compared with 7‐hour sleepers (5.4% vs. 5.0%), the difference was not statistically significant after adjusting for potential confounders (OR = 1.11 [95% CI: 0.91‐1.35]). However, among participants without central obesity at baseline, short sleepers had a higher incidence of central obesity than 7‐hour sleepers (34.5% vs. 32.5%) and a 13% increase in the risk for developing central obesity (OR = 1.13 [95% CI: 1.00‐1.28]) (Table 2). In the sensitivity analyses with further exclusions, the results did not change (data not shown).

## Discussion

Based on the large prospective cohort of the China Kadoorie Biobank, the current study found that short sleep duration was associated with higher risk for gaining weight ≥ 5 kg, especially among short sleepers who were physically inactive, after accounting for sociodemographic, lifestyle, and health status factors. Short sleep duration was also a risk factor for central obesity but not for general obesity.

According to a systematic review of longitudinal studies on sleep duration and subsequent weight gain, the results of adult studies have been less consistent compared with the studies conducted in children 30. The results of the current study are in agreement with studies 15, 16, 24 that reported a significantly elevated risk of gaining weight ≥ 5 kg among participants who were habitual short sleepers. However, unlike studies by Lopez‐Garcia et al. 15 and Chaput et al. 16, we did not observe a significant association between long sleep duration and weight gain. In addition, previous studies have suggested that the association was more prominent in young adults 16, while the present study observed similar impacts across different age groups, which was in line with the results from a large prospective study in the United States 10. Such a discrepancy might be due to the difference in study population, as the current study excluded participants with common comorbidities (e.g., cancer, coronary heart diseases, chronic obstructive pulmonary disease) at baseline to reduce the confounding caused by baseline comorbidities. Also, our results showed that physical activity level modified the association between sleep duration and weight gain. Short sleepers who were physically inactive had a 34% increase in gaining weight ≥ 5 kg. Strong evidence has suggested that physical activity is necessary to prevent weight gain, even more so for those with higher risk for obesity 31. Therefore, being physically inactive might have exacerbated weight control for those short sleepers who were already at higher risk for gaining weight.

Also, a significant negative association was observed between sleep duration with central obesity in relatively healthy adults who were free of common comorbidities but not with general obesity, which suggested that the weight gain is more likely to accumulate on the abdomen rather than whole body. A meta‐analysis including 21 cross‐sectional studies summarized the relationship of short sleep duration with WC 22, but evidence from prospective studies has been sparse. A computed tomography‐based study 32 that included 1,107 participants showed that sleep duration ≤ 5 hours was associated with a 13‐cm^2^ and 42‐cm^2^ higher volume of visceral adipose tissue and subcutaneous adipose tissue among participants younger than 40 years old; similar results were reported among women younger than 40 years old who were habitual short sleepers 14. Moreover, Chaput et al. 33 studied 293 adults who increased sleep duration from ≤ 6 hours to 7 to 8 hours and observed a subsequent reduction in visceral adipose tissue.

Several potential physiological pathways have been suggested for short sleep duration and weight gain. First, both clinical trials and observational studies have suggested shorter sleep increases energy or caloric intake 34, 35 by lowering one’s resistance to unhealthy diet habits, such as high‐caloric and high‐fat food 36, 37, or by downregulating leptin levels and upregulating ghrelin levels to reduce satiety and to increase appetite 38, 39. Second, insufficient sleep might also be associated with lower energy expenditure. Short sleepers are more likely to have lower physical activity levels 40 and resting metabolic rate 41. However, the causal relationship between short sleep duration and weight gain remains uncertain, as a recent Mendelian randomization study failed to link sleep duration with obesity in adults, although some effects were observed in children 42. Further research is needed to confirm the causal relationship between short sleep duration and obesity risk.

To the best of our knowledge, the present study is the most extensive prospective design with objective measurements on obesity, which comprehensively controlled for potential confounders and applied strict exclusion criteria to avoid reverse causality. However, some limitations should be considered. First, sleep duration was self‐reported, which was moderately related to that measured by the objective method polysomnography 43. However, it showed a good reproducibility according to our analysis among those who had repeated measures within a year (*n* = 926, intraclass correlation coefficient: 0.78) 44. Second, sleep quality was not comprehensively assessed by validated scale. However, the association remained in our sensitivity analysis that excluded those who reported any sleep difficulties, which consisted of the main components of sleep quality. Nonetheless, it was suggested that sleep difficulties, such as insomnia, are associated with obesity only when coupled with short sleep duration 45. Third, although we controlled for many lifestyle factors, residual confounding may still exist because of the way covariates were categorized.

In conclusion, the present study provided prospective evidence that short sleep duration (≤ 6 hours) was associated with weight gain ≥ 5 kg and a higher risk for developing central obesity, especially among participants who were physically inactive. Given the trend of sleeping less in the modern society and the high prevalence of central obesity in the Chinese population, the link of sleep with obesity needs to be recognized, and importance of sleep intervention in the context of weight management needs to be underscored.

## References

[1] World Health Organization . Obesity and overweight. https://www.who.int/en/news-room/fact-sheets/detail/obesity-and-overweight. Updated February 16, 2018.

[2] GBD 2017 Risk Factor Collaborators . Global, regional, and national comparative risk assessment of 84 behavioural, environmental and occupational, and metabolic risks or clusters of risks for 195 countries and territories, 1990‐2017: a systematic analysis for the Global Burden of Disease Study 2017. Lancet 2018;392:1923‐1994. 30496105 10.1016/S0140-6736(18)32225-6 PMC6227755

[3] Golem DL , Martin‐Biggers JT , Koenings MM , Davis KF , Byrd‐Bredbenner C . An integrative review of sleep for nutrition professionals. Adv Nutr 2014;5:742‐759. 25398735 10.3945/an.114.006809 PMC4224209

[4] Cournot M , Ruidavets JB , Marquie JC , et al. Environmental factors associated with body mass index in a population of Southern France. Eur J Cardiovasc Prev Rehabil 2004;11:291‐297. 15292762 10.1097/01.hjr.0000129738.22970.62

[5] Doo H , Chun H , Doo M . Associations of daily sleep duration and dietary macronutrient consumption with obesity and dyslipidemia in Koreans: a cross‐sectional study. Medicine (Baltimore) 2016;95:e5360. doi:10.1097/MD.0000000000005360 27828860 PMC5106066

[6] Ko GT , Chan JC , Chan AW , et al. Association between sleeping hours, working hours and obesity in Hong Kong Chinese: the 'better health for better Hong Kong' health promotion campaign. Int J Obes (Lond) 2007;31:254‐260. 16718283 10.1038/sj.ijo.0803389

[7] Moreno CR , Louzada FM , Teixeira LR , et al. Short sleep is associated with obesity among truck drivers. Chronobiol Int 2006;23:1295‐1303. 17190714 10.1080/07420520601089521

[8] Ohayon MM , Vecchierini MF . Normative sleep data, cognitive function and daily living activities in older adults in the community. Sleep 2005;28:981‐989. 16218081

[9] Singh M , Drake CL , Roehrs T , Hudgel DW , Roth T . The association between obesity and short sleep duration: a population‐based study. J Clin Sleep Med 2005;1:357‐363.17564401

[10] Xiao Q , Arem H , Moore SC , Hollenbeck AR , Matthews CE . A large prospective investigation of sleep duration, weight change, and obesity in the NIH‐AARP Diet and Health Study cohort. Am J Epidemiol 2013;178:1600‐1610. 24049160 10.1093/aje/kwt180 PMC3842900

[11] Kobayashi D , Takahashi O , Deshpande GA , Shimbo T , Fukui T . Association between weight gain, obesity, and sleep duration: a large‐scale 3‐year cohort study. Sleep Breath 2012;16:829‐833. 21892668 10.1007/s11325-011-0583-0

[12] Nishiura C , Hashimoto H . A 4‐year study of the association between short sleep duration and change in body mass index in japanese male workers. J Epidemiol 2010;20:385‐390. 20699599 10.2188/jea.JE20100019 PMC3900833

[13] Chien MY , Wang LY , Chen HC . The relationship of sleep duration with obesity and sarcopenia in community‐dwelling older adults. Gerontology 2015;61:399‐406. 25721246 10.1159/000371847

[14] Theorell‐Haglow J , Berglund L , Berne C , Lindberg E . Both habitual short sleepers and long sleepers are at greater risk of obesity: a population‐based 10‐year follow‐up in women. Sleep Med 2014;15:1204‐1211. 25113417 10.1016/j.sleep.2014.02.014

[15] Lopez‐Garcia E , Faubel R , Leon‐Munoz L , Zuluaga MC , Banegas JR , Rodriguez‐Artalejo F . Sleep duration, general and abdominal obesity, and weight change among the older adult population of Spain. Am J Clin Nutr 2008;87:310‐316. 18258619 10.1093/ajcn/87.2.310

[16] Chaput JP , Despres JP , Bouchard C , Tremblay A . The association between sleep duration and weight gain in adults: a 6‐year prospective study from the Quebec Family Study. Sleep 2008;31:517‐523. 18457239 10.1093/sleep/31.4.517 PMC2279744

[17] Zhang S , Li L , Huang Y , Chen K . Meta‐analysis of prospective cohort studies about sleep duration and risk of weight gain and obesity in adults [in Chinese]. Zhonghua Liu Xing Bing Xue Za Zhi 2015;36:519‐525. 26080646

[18] Lauderdale DS , Knutson KL , Rathouz PJ , Yan LL , Hulley SB , Liu K . Cross‐sectional and longitudinal associations between objectively measured sleep duration and body mass index: the CARDIA Sleep Study. Am J Epidemiol 2009;170:805‐813. 19651664 10.1093/aje/kwp230 PMC2765362

[19] Vgontzas AN , Fernandez‐Mendoza J , Miksiewicz T , et al. Unveiling the longitudinal association between short sleep duration and the incidence of obesity: the Penn State Cohort. Int J Obes (Lond) 2014;38:825‐832. 24100421 10.1038/ijo.2013.172 PMC3954466

[20] Stranges S , Cappuccio FP , Kandala NB , et al. Cross‐sectional versus prospective associations of sleep duration with changes in relative weight and body fat distribution: the Whitehall II Study. Am J Epidemiol 2008;167:321‐329. 18006903 10.1093/aje/kwm302 PMC3206317

[21] Janssen I , Katzmarzyk PT , Ross R . Waist circumference and not body mass index explains obesity‐related health risk. Am J Clin Nutr 2004;79:379‐384. 14985210 10.1093/ajcn/79.3.379

[22] Sperry SD , Scully ID , Gramzow RH , Jorgensen RS . Sleep duration and waist circumference in adults: a meta‐analysis. Sleep 2015;38:1269‐1276. 25581918 10.5665/sleep.4906 PMC4507732

[23] Chen Z , Lee L , Chen J , et al. Cohort profile: the Kadoorie Study of Chronic Disease in China (KSCDC). Int J Epidemiol 2005;34:1243‐1249. 16131516 10.1093/ije/dyi174

[24] Chen Z , Chen J , Collins R , et al. Kadoorie Biobank of 0.5 million people: survey methods, baseline characteristics and long‐term follow‐up. Int J Epidemiol 2011;40:1652‐1666. 22158673 10.1093/ije/dyr120 PMC3235021

[25] Lyytikainen P , Rahkonen O , Lahelma E , Lallukka T . Association of sleep duration with weight and weight gain: a prospective follow‐up study. J Sleep Res 2011;20:298‐302. 21199039 10.1111/j.1365-2869.2010.00903.x

[26] Yanovski SZ , Yanovski JA . Obesity. N Engl J Med 2002;346:591‐602. 11856799 10.1056/NEJMra012586

[27] Chen C , Lu FC . The guidelines for prevention and control of overweight and obesity in Chinese adults. Biomed Environ Sci 2004;17(suppl 1):1‐36. 15807475

[28] Alberti KG , Zimmet P , Shaw J ; IDF Epidemiology Task Force Consensus Group . The metabolic syndrome‐‐a new worldwide definition. Lancet 2005;366:1059‐1062. 16182882 10.1016/S0140-6736(05)67402-8

[29] Hirshkowitz M , Whiton K , Albert SM , et al. National Sleep Foundation's sleep time duration recommendations: methodology and results summary. Sleep Health 2015;1:40‐43. 29073412 10.1016/j.sleh.2014.12.010

[30] Magee L , Hale L . Longitudinal associations between sleep duration and subsequent weight gain: a systematic review. Sleep Med Rev 2012;16:231‐241. 21784678 10.1016/j.smrv.2011.05.005 PMC3202683

[31] Swift DL , Johannsen NM , Lavie CJ , Earnest CP , Church TS . The role of exercise and physical activity in weight loss and maintenance. Prog Cardiovasc Dis 2014;56:441‐447. 24438736 10.1016/j.pcad.2013.09.012 PMC3925973

[32] Hairston KG , Bryer‐Ash M , Norris JM , Haffner S , Bowden DW , Wagenknecht LE . Sleep duration and five‐year abdominal fat accumulation in a minority cohort: the IRAS family study. Sleep 2010;33:289‐295. 20337186 10.1093/sleep/33.3.289 PMC2831422

[33] Chaput JP , Bouchard C , Tremblay A . Change in sleep duration and visceral fat accumulation over 6 years in adults. Obesity (Silver Spring) 2014;22:E9‐E12. 24420871 10.1002/oby.20701

[34] Calvin AD , Carter RE , Adachi T , et al. Effects of experimental sleep restriction on caloric intake and activity energy expenditure. Chest 2013;144:79‐86. 23392199 10.1378/chest.12-2829 PMC3707179

[35] St‐Onge MP , Roberts AL , Chen J , et al. Short sleep duration increases energy intakes but does not change energy expenditure in normal‐weight individuals. Am J Clin Nutr 2011;94:410‐416. 21715510 10.3945/ajcn.111.013904 PMC3142720

[36] St‐Onge MP , Wolfe S , Sy M , Shechter A , Hirsch J . Sleep restriction increases the neuronal response to unhealthy food in normal‐weight individuals. Int J Obes (Lond) 2014;38:411‐416. 23779051 10.1038/ijo.2013.114 PMC3883872

[37] Chaput JP , Despres JP , Bouchard C , Tremblay A . The association between short sleep duration and weight gain is dependent on disinhibited eating behavior in adults. Sleep 2011;34:1291‐1267. 21966060 10.5665/SLEEP.1264 PMC3174831

[38] Spiegel K , Tasali E , Penev P , Van Cauter E . Brief communication: sleep curtailment in healthy young men is associated with decreased leptin levels, elevated ghrelin levels, and increased hunger and appetite. Ann Intern Med 2004;141:846‐850. 15583226 10.7326/0003-4819-141-11-200412070-00008

[39] Taheri S , Lin L , Austin D , et al. Short sleep duration is associated with reduced leptin, elevated ghrelin, and increased body mass index. PLoS Med 2004;1:e62. doi:10.1371/journal.pmed.0010062 15602591 PMC535701

[40] Schmid SM , Hallschmid M , Jauch‐Chara K , et al. Short‐term sleep loss decreases physical activity under free‐living conditions but does not increase food intake under time‐deprived laboratory conditions in healthy men. Am J Clin Nutr 2009;90:1476‐1482. 19846546 10.3945/ajcn.2009.27984

[41] Hursel R , Rutters F , Gonnissen HK , Martens EA , Westerterp‐Plantenga MS . Effects of sleep fragmentation in healthy men on energy expenditure, substrate oxidation, physical activity, and exhaustion measured over 48 h in a respiratory chamber. Am J Clin Nutr 2011;94:804‐808. 21795436 10.3945/ajcn.111.017632

[42] Wang J , Li AM , Lam H , Leung GM , Schooling CM . Sleep duration and adiposity in children and adults: observational and mendelian randomization studies. Obesity (Silver Spring) 2019;27:1013‐1022. 31067017 10.1002/oby.22469

[43] Matthews KA , Patel SR , Pantesco EJ , et al. Similarities and differences in estimates of sleep duration by polysomnography, actigraphy, diary, and self‐reported habitual sleep in a community sample. Sleep Health 2018;4:96‐103. 29332687 10.1016/j.sleh.2017.10.011 PMC5771411

[44] Zheng B , Lin LL , Yu CQ , et al. Distributions and associations between duration of sleep, daytime naps and insomnia symptoms among Chinese adults [in Chinese]. Zhonghua Liu Xing Bing Xue Za Zhi 2017;38:452‐456. 28468061 10.3760/cma.j.issn.0254-6450.2017.04.008

[45] Cai GH , Theorell‐Haglow J , Janson C , et al. Insomnia symptoms and sleep duration and their combined effects in relation to associations with obesity and central obesity. Sleep Med 2018;46:81‐87. 29773216 10.1016/j.sleep.2018.03.009

